# The impact of the work organization on individuals’ psychological well-being

**DOI:** 10.3389/fpubh.2025.1677921

**Published:** 2026-01-05

**Authors:** Elisabetta Riccardi, Veruscka Leso, Luca Fontana, Fabio Fusco, Mariagaia Coppola, Daniela Pacella, Ivo Iavicoli

**Affiliations:** 1 Department of Public Health, University of Naples “Federico II”, Naples, Italy; 2 Dipartimento di Sicurezza e Bioetica, Catholic University of Sacred Heart, Rome, Italy; 3 Fondazione Policlinico Universitario A. Gemelli, IRCCS, Rome, Italy

**Keywords:** future of work, occupational health, on-site work, psychological well-being, remote work

## Abstract

**Introduction:**

This study explored the psychological well-being (PWB) of workers engaged in two distinct occupational settings, the transport and instruction sectors, to understand how different work arrangements (on-site, remote, and hybrid) affect workers’ PWB.

**Methods:**

A cross-sectional study was conducted from October 2020 to December 2021, involving employees of a transport company (427), primarily engaged in on-site tasks and University workers (445) involved in remote activities. The PWB was assessed using the Psychological General Well-Being Index.

**Results:**

A general global satisfactory PWB was demonstrated in both groups. However, transport workers showed significantly better results compared to the University personnel, both at the global level (mean ± standard deviation: 87 ± 9 vs. 72 ± 14, respectively) as well as in the anxiety, depression, positive well-being, self-control, general health and vitality sub-dimensions. Age was negatively associated with the PWB among transport workers (*β* = −0.16; *p* < 0.001), while no significant association was demonstrated in the University group. Only in this latter group, the female gender negatively affected the global PWB perception (*β* = −9.0, *p* < 0.001). Working mode, particularly on-site work, was positively associated with the global PWB perception (*β* = 4.8; *p* < 0.042) in university workers.

**Conclusion:**

The type of work organization and individual characteristics may significantly influence workers’ PWB perception. These findings suggest the need for tailored interventions specifically focused on workload and the specific challenges as well as the needs for different occupational groups (e.g., women, older employees, and remote workers) to promote individuals’ well-being in an evolving world of work.

## Introduction

The world of work is experiencing profound transformations due to technological advancements, demographic changes in the workforce, globalization, and evolving organizational models that have been leading the choice to re-arrange working schedules or to modify existing pathways of work. A globally competitive and world-leading industry needs to speed up investment in research and innovation for a 24-h production, refining the way of thinking the “working time.” In parallel, considering the aging and the increasing presence of women among the workforce, organizations must adapt to support individual necessities, in terms also of flexible schedules (1, 2). These transformations became particularly evident during the COVID-19 pandemic period. The “social distancing” and “lock-down” measures of varying severity to curtail COVID-19 spread, inevitably called for a reorganization of the traditional work arrangements and accelerated the adoption of remote and hybrid models, fundamentally reshaping workplace dynamics (3, 4).

Remote work is now a central feature of the modern workplace, as well as hybrid models that combine remote and on-site work. In this scenario, literature evidence demonstrated that an “effective” remote work depends on several factors, including job characteristics and worker competencies. Jobs with clear task criteria, low interdependence, and access to necessary resources facilitate better outcomes for remote workers (5, 6). Additionally, competencies such as self-discipline, adaptability, and communication skills enable individuals to overcome the psychological and structural distance posed by the remote work organization (7, 8). These insights highlight the importance of aligning job design and employee training with remote work demands to ensure the safety, health and well-being of employees.

On the one side, in fact, these models are increasingly favored for their flexibility, allowing employees to balance professional and personal responsibilities (3, 4). On the other side, some challenges may emerge from these types of work organization such as increased ergonomic and psychosocial risks, privacy concerns, and the need for advanced skills. Remote work, in fact, has reshaped the psychosocial dynamics of employment, often intensifying stress-related risks. According to Karasek’s Job Demand-Control model, remote workers may experience high job demands coupled with reduced decision latitude and social support, especially in isolated environments (9, 10). This lack of support can intensify role conflict, heighten anxiety and depression, as well as undermine self-control and vitality. Similarly, Siegrist’s Effort-Reward Imbalance model highlights how diminished recognition and unclear expectations in virtual settings can lead to perceived unfairness (11). The absence of visible effort and feedback may erode motivation, especially when domestic responsibilities interfere with work performance. Isolation and lack of peer support can exacerbate depressive symptoms, especially when combined with low recognition and high demands. From a transactional perspective, the subjective appraisal of remote work stressors—such as digital overload, adequacy if technological resources, constant availability and lack of feedback- can overwhelm coping resources, increasing psychological strain and impacting mental health and productivity (12). Additionally, reduced physical activity—common in remote settings—can impair stress regulation and resilience, diminish positive well-being and perceived mental health compounding the effects of isolation and role strain. Therefore, such profound changes in the work models and organizational structures collectively underscore how the structural and relational shifts of remote work can impact multiple domains of the psychological well-being (PWB) posing mental health to the forefront of occupational concerns (13–15).

To better understand these dynamics within the broader framework of the future of work, the present study explored the PWB of workers, employed in the transport and education sectors, engaged in different types of work arrangement, i.e., on-site or remote work. The primary objective of this study was to evaluate the impact of distinct work arrangements (i.e., on-site, remote, or hybrid) on various domains of the PWB among employees in two occupational sectors characterized by differing organizational structures. Novelty of the study lies in its large sample size, the comparative analysis across sectors, and the use of the Italian-validated Psychological General Well-Being Index (PGWBI) questionnaire, which enables domain-specific assessment of PWB and facilitates benchmarking against national normative data. This investigation yielded critical insights into both risk factors and protective elements associated with each work arrangement, offering actionable recommendations for occupational health practices. These findings align with the Total Worker Health^®^ approach, supporting the development of work environments that are more responsive to individual needs and conducive to enhancing employee well-being.

## Materials and methods

### Study design and participants

A cross-sectional study was carried out between October 2020 and December 2021. Eligible participants (aged ≥18 years) included employees of a public railway and road transport company—such as train drivers, bus drivers, conductors, technicians, and managerial staff—as well as professors, researchers, technical staff, and administrative personnel from a University located in Southern Italy. All were invited to take part in the study.

Participants were recruited from two distinct occupational domains—transportation and education—each represented by a single organization selected through convenience sampling. The choice of these organizations was informed by their willingness to participate, logistical accessibility, and the opportunity they provided to compare diverse work environments and arrangements within contexts marked by differing operational and organizational frameworks. To enable a context-sensitive analysis of remote work experiences, the study intentionally compared two professional settings with markedly different structural, functional, and cultural characteristics: the transport industry and the academic sector. These environments diverge significantly in terms of job nature (e.g., operational versus academic/administrative roles), gender distribution, and task demands. The transportation domain typically involves time-sensitive, location-dependent, and physically coordinated activities, often dominated by male workers and governed by rigid hierarchical structures. Conversely, the University setting is characterized by cognitively intensive, autonomous, and flexible work, with a more balanced or female-skewed gender composition and flatter organizational hierarchies. Such differences suggest distinct profiles in terms of work organization, psychosocial stressors, and the required Knowledge, Skills, Abilities, and Behaviors (KSABs). By integrating these contextual contrasts, the study aims to provide a more nuanced understanding of how varying work arrangements influence PWB across heterogeneous professional landscapes.

As the study was performed during the COVID-19 pandemic period, in fact, the first group of workers was unable to suspend its duties during lockdown or pandemic-related restrictions due to the operational role of the employees, who primarily continued working on-site, maintaining traditional practices with the implementation of necessary safety measures. Conversely, University employees experienced a complete transformation in their work arrangements as they abruptly shifted to remote work or hybrid form of works as their primary mode of operation during the mentioned period of investigation.

Participants were informed about the study’s objectives and procedures and asked to provide informed consent. Personal information, including names, was not collected to ensure confidentiality. The cross-sectional study protocol received approval from the Ethics Committee of the University of Naples “Federico II” (approval ref. No. 278/20) on Month 10/2020. Respondents gave written consent and signature before completing questionnaires.

### Measurement of PWB

PWB was assessed using the Italian-validated version of the PGWBI questionnaire (16) that was self-administered during the health surveillance medical examinations to the workers who provided their consent to be enrolled in the research project. The PGWBI tool is a 22-item self-reported questionnaire aimed at measuring the subjective well-being or distress referred to the last 4 weeks. Six different dimensions are explored: anxiety (ANX), depressed mood (DEP), positive well-being (PoWB), self-control (SC), general health (GH), and vitality (VT). A 6-point Likert scale (from 0 to 5) was used for the evaluation of each item, and consequently, the global score including all the specific areas could reach a maximum of 110 points. Higher scores indicate a better PWB, and in greater detail, overall scores < 54 points, between 55 and 65, and between 66 and 100 indicate severe distress, moderate distress, and “no distress” or positive PWB, respectively. Dimensions of direction are described by a “positive option” with a high score and a “bad option” with a low score, and therefore, a high score in ANX and in DEP indicates low anxiety and low depressed mood, while higher scores in PW, SC, GH, and VT denote high positive well-being, self-control, good health perception, and vitality, respectively. The results obtained for each dimension of the PGWBI in the two groups of workers were compared with the normative data of the Italian population, specifically with the weighted reference values for the male and female population (*n* = 1,129) reported by Grossi et al. (17). Furthermore, both the normative data of the Italian population and the data collected from our examined worker groups were analysed and adjusted following the calculations and evaluation procedures outlined in the scaling and scoring guidelines of the PGWBI proposed by Dupuy (18), thus ensuring methodological consistency and comparability between the datasets.

### Statistics

Data were presented as frequency (percentages) for the categorical variables, while they were indicated as mean ± standard deviation for continuous variables. Differences between means were performed using the Student’s t test or the Mann–Whitney U test, as appropriate. Associations between the considered factors and the outcome variables (PGWBI subscale and total scale scores) were evaluated using univariable linear regression models. If multiple variables (>2) were significant at the univariable regression analysis were then included in a multivariable linear regression model. Where applicable, Bonferroni correction for multiple comparisons was applied. For every regression model, assumptions of linearity, homoskedasticity and normality of residuals were tested visually inspecting Q-Q plots of the residuals and using the Breusch-Pagan test. In order to investigate the potential need for stratified analyses, we have run separate regression models on the whole sample (including both workers’ groups) adding as predictors, respectively in separate models: the interaction between the work group and age, the interaction between the work group and sex and finally the interaction between work group and work mode. We have run all of these models for all of the PGWBI scores (total and each subdomain). For all analyses, the significance level was set at a *α* 0.05 (two-sided). All analyses were performed using R Statistical Software, version 4.0.3.

## Results

### Socio-demographic and occupational data

Of the 980 workers who were invited to participate, 872 (89%) completed the survey, 427 and 445 from the transport company and the University personnel, respectively. Almost the totality of the transport workers were males (*n* = 398 out of 415 respondents, 96%), while among the University employees, a lower percentage of males was present (*n* = 243 out of 444 respondents, 55%) (*p* < 0.001). The two groups of workers showed a comparable age, with a mean ± standard deviation (SD) age of 52 ± 10 and 53 ± 9 years, for transport and University workers, respectively (Table 1). Concerning the specific job tasks of transport workers, across the 378 respondents, most were train drivers (*n* = 172, 46%), followed by conductors and bus drivers (*n* = 93, 25%), coordination and management workers (*n* = 67, 18%), technical and maintenance operators (*n* = 46, 12%). Given the nature of the work activity and the specificity of the tasks for the most transport workers the predominant working mode was on-site (*n* = 352 out of 372 respondents, 95%). Within the group of 437 University workers respondents, the prevailing job categories included professors (*n* = 207, 46.5%) and technicians/administrative staff (*n* = 205, 46%), followed by researchers (*n* = 25, 5.5%). Among the 438 respondents, these employees were primarily engaged in remote working, exclusively (*n* = 177, 40%) or in a mixed mode with some days on-site (*n* = 218, 50%), while only few of them worked entirely on-site (*n* = 43, 9.8%). The differences evidenced by these demographic aspects justify the need for separating analyses by workers’ group.

**Table 1:** Socio-demographic and occupational characteristics of transport and University workers recruited in the study. Significant values are marked with bold type.

Characteristics	*N*	Transport workers, *N* = 427	University workers, *N* = 445	*p*-value
Gender	859			**<0.001**
M		398 (96%)	243 (55%)	
F		17 (4.1%)	201 (45%)	
NA		12	1	
Age	776	52 (10)	53 (9)	0.173
NA		63	33	
Children	811			**<0.001**
No		49 (13%)	112 (26%)	
Yes		329 (87%)	321 (74%)	
NA		49	12	
Work organization	810			**<0.001**
Remote		8 (2.2%)	177 (40%)	
On-site		352 (95%)	43 (9.8%)	
Mixed		12 (3.2%)	218 (50%)	
NA		55	7	
Role	815			
Train drivers		172 (46%)		
Conductor and bus drivers		93 (25%)		
Technical and maintenance operators		46 (12%)		
Coordination and management workers		67 (18%)		
Professors			207 (47%)	
Technicians/administrative staff			205 (47%)	
Researchers			25 (5.7%)	

Significant values are marked with bold type.

### PWB perception: global score

The mean ± SD of the PGWBI global scores for the transport and University workers were 87 ± 9 and 72 ± 14, respectively (Table 2). These results indicated, in both groups, a condition of “no distress,” corresponding to a global satisfactory PWB. However, when a comparison was performed with the national PWB data, and between the two enrolled groups, the perceived PWB in the transport workers was significantly higher compared to both the general population (*p* < 0.001) and the University workers (*p* < 0.001), while no significant difference could be determined between the University and the general population (*p* = 0.441).

**Table 2:** Psychological General Well-Being Index (PGWBI) global and sub-domain scores in transport and University workers and comparison with PGWBI values observed in the Italian general population.

PGWBI	Transport workers *N* = 427	University workers *N* = 445	*p*-value between transport and University workers	Italian population (reference) *N* = 1,129	Transport workers *N* = 427	*p*-value between transport workers and the reference population	Italian population (reference) *N* = 1,129	University workers *N* = 445	*p*-value between University worker and the reference population
Anxiety	89 (12)	70 (18)	**<0.001**	69 (20)	89 (12)	**<0.001**	69 (20)	70 (18)	0.441
Depression	95 (9)	86 (13)	**<0.001**	83 (17)	95 (9)	**<0.001**	83 (17)	86 (13)	**<0.001**
Positive well-being	77 (14)	61 (17)	**<0.001**	69 (20)	77 (14)	**<0.001**	69 (20)	61 (17)	**0.047**
Self control	90 (13)	81 (41)	**<0.001**	79 (18)	90 (13)	**<0.001**	79 (18)	81 (41)	0.248
General health	88 (11)	77 (15)	**<0.001**	74 (20)	88 (11)	**<0.001**	74 (20)	77 (15)	**0.001**
Vitality	83 (11)	66 (16)	**<0.001**	67 (20)	83 (11)	**<0.001**	67 (20)	66 (16)	0.300
Global	87 (9)	72 (14)	**<0.001**	71 (20)	87 (9)	**<0.001**	71 (20)	72 (14)	0.185

Higher scores indicate a better PWB. Overall scores < 54 points, between 55 and 65, and between 66 and 100 indicate severe distress, moderate distress, and “no distress” or positive PWB, respectively. Significant values are marked with bold type.

In line with this positive PWB global perception, the transport workers, also in the analyses of the ANX and DEP domains, showed mean ± SD levels (89 ± 12 and 95 ± 9, respectively) indicative for a non-distressed condition, and significantly better compared to data reported for the general population (69 ± 20 and 83 ± 17, respectively) and the University workers (70 ± 18 and 86 ± 13, respectively) (*p* < 0.001), although all the values of this latter group remained in a positive range and significantly higher compared to the general population for the DEP area (*p* < 0.001).

Similarly, in other domains, SC (90 ± 13), GH (88 ± 11), and VT (83 ± 11) transport workers showed positive and significantly higher mean ± SD levels compared to the Italian general population and the University reference group (*p* < 0.001) (Table 2). This latter one, on the other side, showed values significantly higher compared to the reference general population only in the GH domain (77 ± 15 vs. 74 ± 20, respectively) (*p* < 0.001), and significantly lower in the PoWB (61 ± 17 vs. 69 ± 20, respectively) (*p* < 0.001).

### PWB perception: subgroup analysis

#### Transport workers group

**Figure 1:**
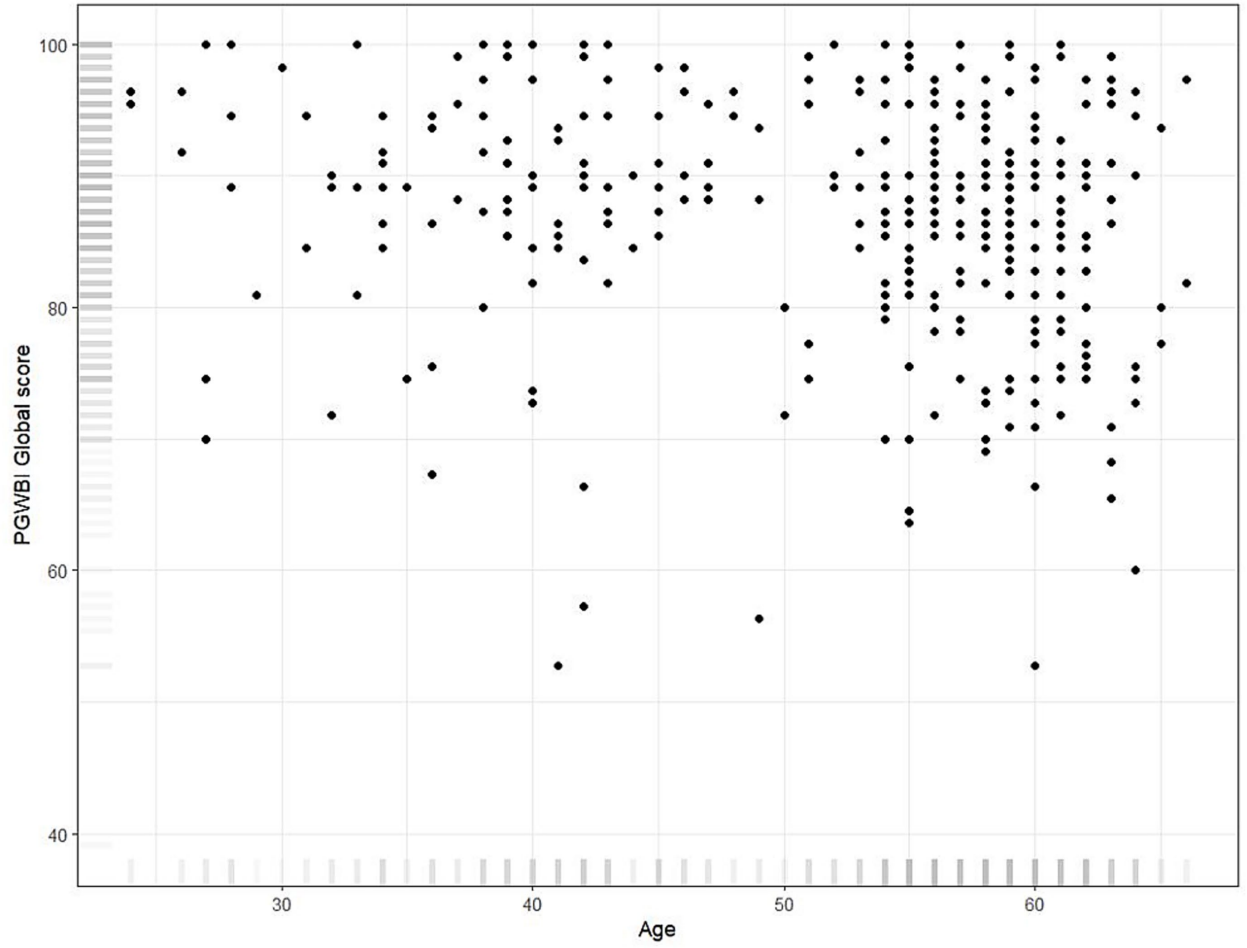
Scatterplot displaying the relationship between age and PGWBI global score in the transport workers group.

The evaluation of the global PWB across occupational subgroups (Table 3) revealed that younger transport workers reported the highest levels of well-being. This finding is supported by the linear analysis, which showed a significant negative association between age and the global PGWBI score (*β* = −0.16, *p* < 0.001). This finding indicates a consistent trend in which younger employees tend to report more favorable psychological outcomes. This may be attributed to higher baseline levels of VT, greater adaptability to changing circumstances, and fewer caregiving responsibilities compared to their older counterparts. Figure 1 displays the relationship between age and PGWBI global score. Furthermore, age was negatively associated with nearly all PGWBI subdomains. Specifically, younger employees experienced lower levels of ANX (*β* = −0.19, *p* = 0.001) and DEP (*β* = −0.10, *p* = 0.023), alongside higher scores in PoWB (*β* = −0.28, *p* < 0.001), GH perception (*β* = −0.17, *p* = 0.002), and VT (*β* = −0.16, *p* = 0.006) (Table 4) suggesting a consistent trend of age-related vulnerability.

**Table 3:** Variables affecting the global psychological well-being perception in the transport workers at univariable linear regressions.

Characteristics	*N*	Beta	95% CI	*p*-value
Gender	415			
M		—	—	
F		−3.3	−7.8, 1.2	0.148
Age	364	−0.16	−0.25, −0.07	**<0.001**
Children	378			
No		—	—	
Yes		−1.0	−3.7, 1.7	0.451
Work organization	372			
Remote		—	—	
On-site		−2.7	−9.2, 3.9	0.424
Mixed		3.4	−4.9, 12	0.422
Role	378			
Train drivers		—	—	
Conductor and bus drivers		1.8	−0.44, 4.0	0.116
Technical and maintenance operators		0.38	−2.5, 3.2	0.794
Coordination and management workers		−0.66	−3.1, 1.8	0.595

*N* report the sample size on which the analyses were carried out. Significant values are marked with bold type.

**Table 4:** Variables affecting the subdomains of the psychological well-being perception in the transport workers at univariable linear regressions.

Characteristic	*N*	Anxiety			Depression			Positive well-being			General-health			Vitality			Self-control		
		Beta	95% CI	*p*-value	Beta	95% CI	*p*-value	Beta	95% CI	*p*-value	Beta	95% CI	*p*-value	Beta	95% CI	*p*-value	Beta	95% CI	*p*-value
Gender	415																		
M		—	—		—	—		—	—		—	—		—	—		—	—	
F		−1.9	−7.8, 4.0	0.527	−2.9	−7.4, 1.7	0.215	−2.8	−9.7, 4.1	0.423	−0.79	−6.0, 4.5	0.768	−6.5	−12, −1.1	**0.018**	−5.4	−11, 0.67	0.081
Age	364	−0.19	−0.31, −0.06	**0.003**	−0.10	−0.19, −0.01	**0.023**	−0.28	−0.42, −0.14	**<0.001**	−0.17	−0.28, −0.06	**0.002**	−0.16	−0.27, −0.05	**0.006**	−0.01	−0.13, 0.12	0.927
Children	378																		
No		—	—		—	—		—	—		—	—		—	—		—	—	
Yes		−1.1	−4.7, 2.5	0.558	−0.76	−3.5, 2.0	0.589	1.6	−2.5, 5.8	0.441	−1.8	−5.0, 1.4	0.262	−2.0	−5.3, 1.3	0.237	−1.9	−5.7, 2.0	0.336
Work organization	372																		
Remote		—	—		—	—		—	—		—	—		—	—		—	—	
On-site		−3.3	−12, 5.3	0.453	−2.5	−9.3, 4.3	0.468	−3.5	−13, 6.5	0.494	−0.47	−8.0, 7.1	0.902	−0.77	−8.7, 7.1	0.849	−5.0	−14, 3.8	0.267
Mixed		5.5	−5.4, 16	0.320	1.9	−6.7, 11	0.658	6.7	−6.0, 19	0.303	3.3	−6.3, 13	0.496	6.7	−3.4, 17	0.195	−7.2	−18, 4.0	0.207
Role	378																		
Train driver		—	—		—	—					—	—		—	—		—	—	
Conductor and bus driver		1.0	−1.8, 3.8	0.472	1.3	−0.50, 3.1	0.155	4.3	0.89, 7.7	**0.014**	2.9	0.28, 5.5	**0.030**	2.1	−0.70, 4.9	0.141	−1.8	−4.8, 1.2	0.243
Technical and maintenance operator		0.43	−3.1, 4.0	0.813	−1.5	−3.9, 0.79	0.194	4.5	0.12, 9.0	**0.044**	1.5	−1.9, 4.9	0.386	−0.08	−3.7, 3.5	0.964	−2.6	−6.6, 1.3	0.186
Coordination and management workers		−1.0	−4.1, 2.1	0.511	−0.17	−2.2, 1.9	0.869	−1.6	−5.4, 2.2	0.415	−0.23	−3.1, 2.7	0.878	−0.28	−3.4, 2.8	0.858	−1.4	−4.8, 2.0	0.426

*N* reports the sample size on which the analyses were carried out. Significant values are marked with bold type.

Regarding gender, only VT subdomain showed a significant negative association with female gender (*β* = −6.5, *p* = 0.018), suggesting that women reported lower energy and resilience compared to men.

Job task also emerged as a relevant factor: conductors and bus drivers reported better outcomes in GH and PoWB compared to train drivers, with positive associations observed in linear analysis (GH: *β* = 2.9, *p* = 0.030; PoWB: *β* = 4.3, *p* = 0.014). Similarly, technical and maintenance operators showed higher levels of PoWB (*β* = 4.5, *p* = 0.044), possibly reflecting greater task clarity or physical engagement. These differences highlight the importance of role-specific demands and resources in shaping PWB.

Finally, no significant association was found between working mode and global PGWBI scores in this subgroup, suggesting that, within this occupational context, remote versus on-site work may not be a primary determinant of overall PWB.

#### University workers

In the University group, age was not demonstrated to be significantly associated with the PWB perception (*β* = −0.11, *p* < 0.160). However, a significant negative association was identified for the DEP (*β* = −0.16, *p* = 0.033) and PoWB (β = −0.30, *p* = 0.003), suggesting that younger workers could experience lower levels of DEP and a higher level of PoWB domain (*β* = −0.30, *p* = 0.003).

Female gender was significantly associated with lower well-being perception across multiple PGWBI domains. Specifically, female University staff scored 9 points lower on the global PGWBI index (*β* = −9.0, *p* < 0.001), a difference that corresponds to a clinically meaningful shift—potentially moving individuals from a state of positive well-being into mild or moderate distress, based on established PGWBI thresholds. Similarly, higher levels of ANX (*β* = −11.0, *p* < 0.001) and DEP (*β* = −5.9, *p* < 0.001) were observed, alongside reduced scores in PoWB (*β* = −10.0, *p* < 0.001), GH (*β* = −7.3, *p* < 0.001), and VT (*β* = −9.1, *p* < 0.001) (Tables 5, 6).

**Table 5:** Variables affecting the global psychological well-being perception in the University personnel at univariable linear regressions.

Characteristic	*N*	Beta	95% CI	*p*-value
Gender	444			
M		—	—	
F		−9.0	−11, −6.6	**<0.001**
Age	412	−0.11	−0.26, 0.04	0.160
Children	433			
No		—	—	
Yes		−0.36	−3.3, 2.6	0.814
Work organization	438			
Remote		—	—	
On-site		4.8	0.18, 9.4	**0.042**
Mixed		0.76	−2.0, 3.5	0.584
Role	437			
Professors		—	—	
Technicians/administrative staff		−2.9	−5.6, −0.29	**0.030**
Researchers		−0.07	−5.8, 5.7	0.981

*N* reports the sample size on which the analyses were carried out. Significant values are marked with bold type.

**Table 6:** Variables affecting the subdomains of the psychological well-being perception in the university personnel at univariable linear regressions.

Characteristic	*N*	Anxiety			Depression			Positive well-being			General-health			Vitality			Self-control		
		Beta	95% CI	*p*-value	Beta	95% CI	*p*-value	Beta	95% CI	*p*-value	Beta	95% CI	*p*-value	Beta	95% CI	*p*-value	Beta	95% CI	*p*-value
Gender	444																		
M		—	—		—	—		—	—		—	—		—	—		—	—	
F		−11	−14, −7.8	**<0.001**	−5.9	−8.4, −3.5	**<0.001**	−10	−13, −7.1	**<0.001**	−7.3	−9.9, −4.6	**<0.001**	−9.1	−12, −6.2	**<0.001**	−4.8	−13, 2.9	0.224
Age	412	0.00	−0.20, 0.20	0.989	−0.16	−0.31, −0.01	**0.033**	−0.30	−0.49, −0.10	**0.003**	−0.13	−0.30, 0.03	0.104	−0.02	−0.20, 0.16	0.811	0.16	−0.31, 0.63	0.509
Children	433																		
No		—	—		—	—		—	—		—	—		—	—		—	—	
Yes		−0.09	−4.0, 3.8	0.964	−0.63	−3.5, 2.3	0.671	−0.08	−3.8, 3.7	0.964	1.0	−2.1, 4.2	0.520	−1.0	−4.5, 2.5	0.572	−8.3	−17, 0.71	0.071
Work organization	438																		
Remote		—	—		—	—		—	—		—	—		—	—		—	—	
On-site		−0.68	−6.7, 5.3	0.824	5.9	1.5, 10	**0.009**	11	5.0, 16	**<0.001**	2.6	−2.3, 7.6	0.294	4.0	−1.5, 9.5	0.151	3.2	−11, 17	0.652
Mixed		−0.21	−3.8, 3.4	0.910	0.72	−1.9, 3.4	0.593	4.1	0.72, 7.5	**0.018**	−1.1	−4.0, 1.9	0.478	0.71	−2.5, 4.0	0.670	−4.8	−13, 3.5	0.255
Role	437																		
Professors		—	—		—	—		—	—		—	—		—	—		—	—	
Technicians/administrative staff		−0.76	−4.3, 2.7	0.670	−2.6	−5.2, −0.02	**0.048**	−2.5	−5.9, 0.83	0.140	−3.7	−6.5, −0.85	**0.011**	−3.7	−6.9, −0.56	**0.021**	−9.6	−18, −1.6	**0.019**
Researchers		1.4	−6.2, 8.9	0.723	−0.56	−6.1, 5.0	0.844	3.2	−4.0, 10	0.378	−1.1	−7.2, 5.0	0.715	−2.7	−9.5, 4.1	0.438	−5.7	−23, 11	0.514

*N* reports the sample size on which the analyses were carried out. Significant values are marked with bold type.

**Figure 2:**
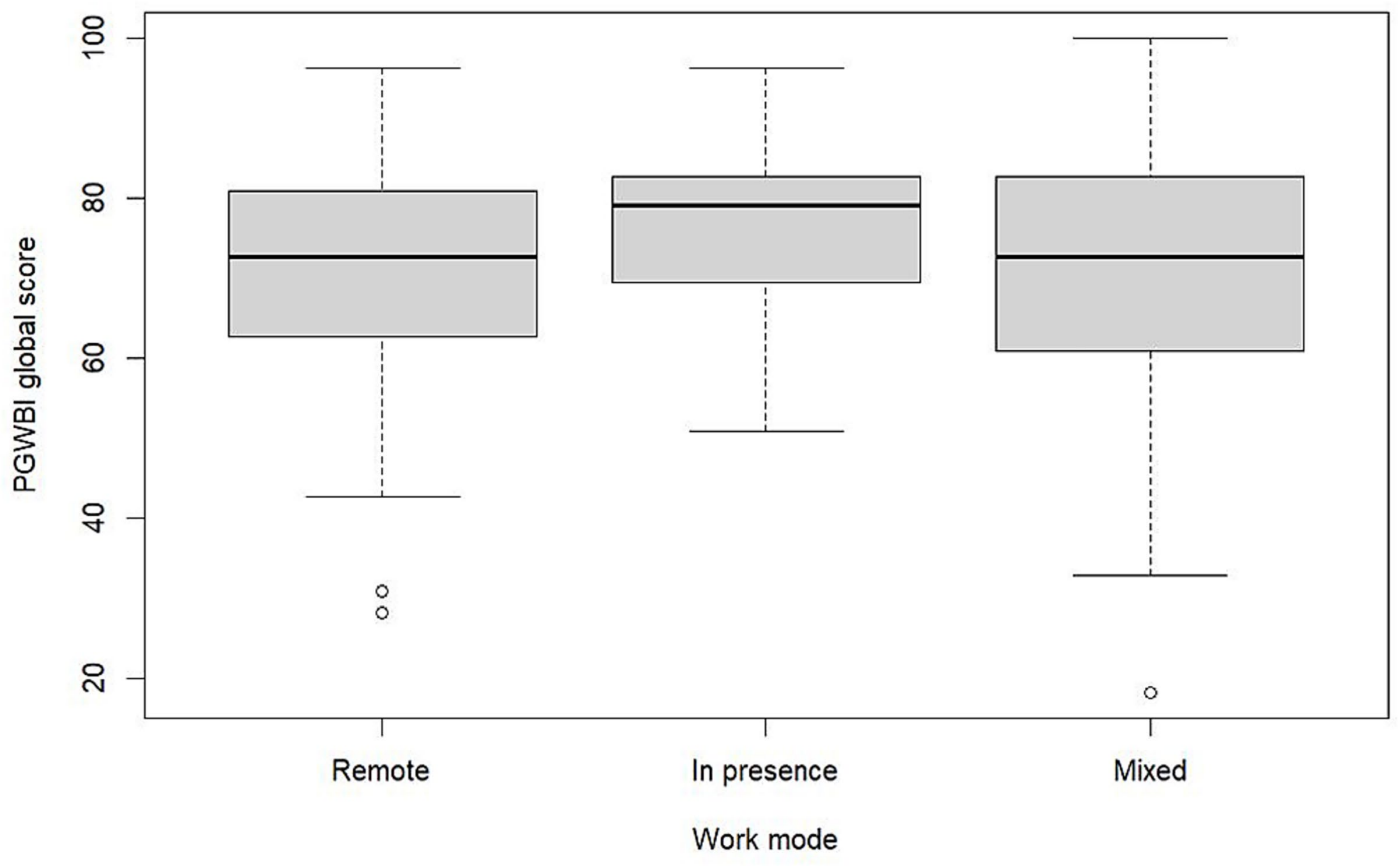
PGWBI global score by work mode in the university staff workers group.

Working mode—and particularly on-site work—was positively associated with global PGWBI (*β* = 4.8, *p* = 0.042). On-site work was also linked to significantly lower levels of DEP (*β* = 5.9, *p* = 0.009) and substantially higher scores in PoWB (*β* = 11.0, *p* < 0.001), suggesting that physical presence at the workplace may offer protective psychosocial benefits. Mixed-mode working was positively associated with higher levels of PoWB compared to exclusive remote work (*β* = 4.1, *p* = 0.018), suggesting that alternating between remote and on-site modalities may offer psychosocial benefits. Figure 2 illustrates the differences in PGWBI global scores by working mode within this subgroup.

Focusing on job roles, being employed as a technician or administrative staff was negatively associated with several PGWBI domains: DEP (*β* = −2.6, *p* = 0.048), SC (*β* = −9.6, *p* = 0.019), GH (*β* = −3.7, *p* = 0.011), VT (*β* = −3.7, *p* = 0.021), and global PGWBI (*β* = −2.9, *p* = 0.030). These effect —particularly the substantial reduction in self-control—may reflect the limited autonomy, repetitive tasks, and lower decision latitude typically associated with these roles, which are known to impact psychological resilience and coping capacity.

Finally, the separate regression models including the complete cohort and considering as predictors of global PGWBI score and subdomain scores, respectively, the interaction between the work group and age, the interaction between the work group and sex and the interaction between work group and work mode revealed no significant interactions. These results highlight that, in our sample, age, sex and work mode affect both types of workers’ scores in the same manner.

## Discussion

This study examined PWB and its subdomains among workers in two distinct organizational settings: a public transport company with primarily on-site work, and a University with remote or hybrid arrangements. The aim was to assess how different work structures influence PWB, particularly during times of organizational transition and exposure to stressors such as the recent COVID-19 pandemic.

The results showed significant differences between the two occupational groups. Transport workers reported higher overall PWB than both the general Italian population and University workers, with better scores across all PWB dimensions. This finding, collected during the COVID-19 pandemic, may reflect the greater stability and sense of purpose experienced by those continuing on-site work, which helped buffer mental health challenges (19, 20). In contrast, University workers faced a sudden shift to remote work without adequate preparation, disrupting routines and requiring rapid adaptation. This often led to isolation, reduced social support, and blurred work-life boundaries (20–24).

During the pandemic, reduced workload in the transport sector—due to fewer journeys and passengers—helped ease stressors like overcrowding, conflict, and contagion risk, temporarily improving well-being (25). The physically active nature of on-site work may have also supported greater VT and GH compared to the sedentary routines of remote workers, which often increased monotony, stress, and ANX (26, 27). For remote employees, despite benefits like reduced commuting and more flexibility, the shift intensified psychological stress, contributing to lower well-being in some areas. This may explain the higher ANX, DEP, and lower VT reported by female empoyees, as well as the poorer PWB among older workers, who showed greater vulnerability and reduced resilience. Gender-related psychosocial factors—such as unequal exposure to caregiving responsibilities, work-life conflict, and access to coping resources—may substantially shape the psychological impact of remote work (28–31). Similarly, older employees may experience poorer PWB due to increased vulnerability, reduced adaptability to technological and organizational changes, and diminished resilience in the face of shifting work demands (32). These age-related challenges can be compounded by health concerns and a greater likelihood of social isolation during remote work periods. Moreover, the intersection of gender and age with occupational roles and organizational expectations can further influence how remote work is experienced and managed, underscoring the need for more inclusive and supportive workplace policies that address the diverse needs of employees across different demographic profiles.

It remains challenging to determine whether the observed differences in PWB are primarily attributable to sector-specific characteristics or to the mode of work (on-site versus remote), as these factors are closely interrelated and may exert overlapping influences. Nonetheless, in University employees, on-site work appears to be associated with more favorable psychological outcomes, potentially reflecting a meaningful shift toward improved well-being when compared to remote modalities. Physical presence in the workplace may offer protective psychosocial benefits, including greater access to social support, clearer role boundaries, and reduced feelings of isolation. These elements are known to play a crucial role in shaping psychological health. Furthermore, mixed-mode working—alternating between remote and on-site arrangements—seems to promote positive well-being, possibly by balancing the autonomy and flexibility of remote work with the interpersonal engagement and structure provided by in-person settings. Additionally, when considered in relation to established PWB benchmarks, diverse occupational job tasks appear to exhibit different vulnerability to distress. This highlights the critical need for targeted support strategies that take into account the specific demands, stressors, and resources associated with different professional roles.

Moreover, other factors may complicate the interpretation of the results and limit the ability to draw definitive conclusions. First of all, the gender distribution varied significantly between the two sectors, which may have introduced a confounding bias, particularly in light of well-documented gender differences in psychological responses to remote work, work-life balance, and caregiving responsibilities (28–31). These disparities could have amplified or masked the true effects of work modality or sectoral context on PWB. Additionally, while the selected samples provide valuable insights into sector-specific dynamics, it is important to acknowledge that they may not fully represent the broader workforce within each sector. The reliance on single-company sampling limits the generalizability of the findings, as organizational culture, policies, and work environments may differ significantly across companies within the same industry. Therefore, caution is warranted when extrapolating the results beyond the participating organizations.

Furthermore, the use of the PGWBI, while a validated and widely used instrument for assessing general PWB, may not be sufficiently sensitive to capture the nuanced and occupation-specific stressors and psychosocial demands inherent in different work environments. As such, certain dimensions of work-related distress or satisfaction may have been underrepresented, potentially limiting the depth and specificity of the findings. In this context, given that the relationship between work modality, gender, and age may follow a non-linear pattern and be influenced by latent perceptual factors, adopting hybrid modeling approaches could offer valuable analytical advantages. For instance, the integration of structural equation modeling with artificial neural networks (SEM–ANN) or similar hybrid frameworks may enhance the precision of causal inference and facilitate the identification of complex, non-obvious predictors linking organizational perceptions to individual outcomes (33). Such models are particularly well-suited to capturing intricate interactions and mediating effects that traditional linear methods might overlook.

To support worker well-being across diverse organizational settings, the TWH framework promotes integrating occupational safety with broader health-promoting policies and practices (34–37). Leadership is encouraged to adopt strategies tailored to sector-specific needs, particularly in addressing remote work challenges. Key actions include: (a) redesigning work organization and support systems to help employees manage workload and reduce risks; (b) providing preparatory training before introducing new modalities. Structured onboarding should cover technology use, time management, and social connection to facilitate adaptation and cohesion. Leadership should also: (c) conduct regular check-ins to monitor well-being (e.g., via PGWBI) and connect with occupational health services; (d) encourage virtual social interaction; (e) support work-life balance through flexible, gender-sensitive policies; and (f) implement age-sensitive measures that promote active ageing and align job roles with workers’ strengths. These strategies underscore the role of proactive leadership in fostering remote work environments that are both productive and health-supportive.

In alignment with leadership-driven strategies, worker engagement is a vital element of the TWH approach, especially in remote work contexts. A participatory model enables employees to shape health-promoting practices, enhancing well-being and organizational resilience. Core actions include promoting physical activity, reducing sedentary behavior, fostering psychosocial health through social connection, and enforcing boundaries between work and personal life—such as the right to disconnect. These measures help mitigate remote work’s adverse effects and support health promotion. Encouraging proactive attitudes and confidence in work ability supports active ageing and employability. Additional initiatives like resilience workshops, mental health counselling, and targeted programs addressing safety and well-being further strengthen the TWH framework across occupational settings.

Our results and the consequent actionable strategies outlined for leadership and worker engagement offer practical guidance for workplace practitioners seeking to translate research findings into effective organizational policies. For policymakers, these insights can inform the development of national workplace standards that promote health, safety, and productivity across sectors. For example, sector-specific guidance for Universities could incorporate structured onboarding for hybrid work, policies supporting the right to disconnect, and age- and gender-sensitive measures that foster inclusion and long-term employability. Embedding such evidence-based practices into national frameworks would support healthier work environments, and enhance workforce resilience at scale. Integrating such initiatives into remote work settings can yield measurable public health benefits. These include reduced absenteeism, improved productivity, and lower incidence of stress-related conditions. Scaling such interventions across sectors may contribute to healthier, more resilient working populations and reduced healthcare burden in line with literature evidence that higher well-being correlates with better work performance and long-term employability (38, 39).

Despite the valuable findings, further research is needed to address the study’s limitations. Its cross-sectional design prevents causal inference between work features and PWB. Longitudinal studies, experimental follow-ups, or intervention-based designs—especially when paired with advanced analytical techniques such as hybrid modeling (e.g., the integration of structural equation modeling with artificial neural networks, SEM–ANN) (33)—could more effectively capture complex predictors and enhance the robustness of causal inference. Where feasible, statistical control for sectoral imbalance would also strengthen the robustness of future results. Additionally, research should focus on maintaining the desired a-priori power even after subgroup or stratified analyses. Overall, this may also help to overcome the bias introduced by the COVID-19 pandemic period during which the study was performed, that inevitably functioned as a confounding factor in the relationship between the mode of work and the PWB. Further research should aim to include a more diverse and representative sample across multiple companies to enhance external validity and better capture sector-wide trends. Moreover, future investigation should correlate “objective-sentinel events” in the workplace, such as absenteeism and presenteeism, unused holidays, department or job task changes, visits to the occupational physicians upon request with the individual, subjective, self-perceived PWB assessment that could have been biased by the volunteering recruitment of workers and the employment of the single-PGWBI tool for the assessment. Additionally, also other features, such as the length of service, personality characteristics, lifestyles and personal history should be evaluated for their possible impact on the PWB.

In conclusion, this study highlights how PWB is shaped by the interaction of work organization, individual factors, and broader societal conditions. Transport workers showed greater well-being during the pandemic, likely due to stable routines and physical engagement, while University staff faced challenges linked to remote work transitions. These findings support the need for tailored interventions and policies—guided by the TWH principles—to strengthen psychosocial resources and promote resilience. To improve outcomes, organizations should invest in tailored onboarding, flexible scheduling, and psychosocial support. Implementing such strategies can help organizations sustain productivity while safeguarding and promoting employee well-being in evolving work environments.

## Data Availability

The raw data supporting the conclusions of this article will be made available by the authors, without undue reservation.
